# Thermal Water Applications in the Treatment of Upper Respiratory Tract Diseases: A Systematic Review and Meta-Analysis

**DOI:** 10.1155/2014/943824

**Published:** 2014-06-01

**Authors:** Sarah Keller, Volker König, Ralph Mösges

**Affiliations:** Institute of Medical Statistics, Informatics and Epidemiology (IMSIE), University Hospital of Cologne, 50924 Cologne, Germany

## Abstract

*Background*. Thermal water inhalations and irrigations have a long tradition in the treatment of airway diseases. Currently there exists no systematic review or meta-analysis on the effectiveness of thermal water treatment in upper respiratory tract diseases. *Methods*. A systematic search in the databases of MEDLINE, EMBASE, CENTRAL, ISI Web of Science, and MedPilot was accomplished. *Results*. Eight evaluable outcome parameters from 13 prospective clinical studies were identified for 840 patients. Mucociliary clearance time improves significantly (*P* < 0.01) for the pooled thermal water subgroup and the sulphurous subgroup after 2 weeks (−6.69/minutes) and after 90 days (−8.33/minutes), not for isotonic sodium chloride solution (ISCS). Nasal resistance improved significantly after 2 weeks (Radon, ISCS, and placebo), after 30 days (sulphur and ISCS), and after 90 days (sulphur). Nasal flow improved significantly with the pooled thermal water, radon alone, and ISCS subgroups. For the IgE parameter only sulphurous thermal water (*P* < 0.01) and ISCS (*P* > 0.01) were analyzable. Adverse events of minor character were only reported for sulphurous treatment (19/370). *Conclusion*. Thermal water applications with radon or sulphur can be recommended as additional nonpharmacological treatment in upper airway diseases. Also in comparison to isotonic saline solution it shows significant improvements and should be investigated further.

## 1. Introduction

Upper airway diseases compass acute and chronic conditions. In this study, we focus on recurrent upper respiratory tract infections (RURT), allergic rhinitis (AR), nonallergic rhinitis (NAR), and acute and chronic rhinosinusitis (ARS/CRS) with and without nasal polyps. These disorders are extremely common and present in all ages, all ethnic populations, and all countries [1]. Apart from their high socioeconomic burden [2], “comorbidities are common and increase the complexity of the management and costs” [1].

Rhinitis is a symptomatic inflammation of the nasal mucosa including nasal symptoms like rhinorrhea, nasal obstruction, nasal itching, and sneezing [3]. The most common form of noninfectious rhinitis is AR with immunoglobulin E- (IgE-) mediated immune response after allergen exposure [1]. Nonallergic rhinitis shows periodic or perennial symptoms, which are not IgE-dependent such as infectious or vasomotor rhinitis [4]. Infectious rhinitis has either viral, bacterial, or other infectious agents origin [3] and affects millions of people annually [5].

Rhinitis and sinusitis mostly coexist and have been proposed as* rhinosinusitis *[6]. The European Position Paper on Rhinosinusitis and Polyps* EPOS* 2012 [7] defines rhinosinusitis as an inflammation of the nose and paranasal sinuses characterised by two or more symptoms, one of which should be either nasal blockage/obstruction/congestion or nasal discharge. Further, either endoscopic or CT proof is obligatory. Acute and chronic rhinosinusitis are distinguished in length of illness and grade of decay of symptoms. It is characterised by a duration of more than 12 weeks without complete resolution of symptoms and affects approximately 5–15% of the general population [7–9].

State-of-the-art documents ARIA [3] and EPOS [7] provide evidence-based treatment guidelines where “anti-inflammatory medication represents the first-line treatment” [10]. Along with steroid/pharmacological treatment, EPOS [7] recommends nasal saline irrigation as additional first-line treatment in acute and chronic rhinosinusitis and after sinonasal surgery [7]. Some reviews also state nasal irrigation as adjunctive treatment for allergic rhinitis, acute upper respiratory tract infections, and rhinitis of pregnancy [11–13]. Data for nasal inhalation is limited; EPOS [7] mentions inhalation treatment only in acute rhinosinusitis without evidence. Other guidelines from the German Society of Otorhinolaryngology have an open suggestion for inhalation in rhinosinusitis for symptomatic relief [14].

The current treatment regimens for CRS and AR are effective in the majority of patients, but there are a number of patients still suffering from symptoms [10]. Especially under chronic conditions with long-term drug consumption like glucocorticosteroids, many patients hesitate to take medicine. A publication by Kaschke points out that 64% of AR patients have steroid phobia [15]. It is observed that more and more patients inquire about nonpharmacological therapy approaches in the treatment of rhinosinusitis [16]. Possible approaches besides the proven saline irrigation could be the use of thermal water irrigations and inhalations.

Concerning medical spending, a study by Bhattacharyya points out that the annual costs for medication to treat CRS with intranasal steroids, nonsedating antihistamines, and antibiotic therapy were $213, $227, and $335 in 2003 [2]. Averaging the annual cost of sinus medications including over-the-counter remedies, nasal steroid sprays, and antibiotics, the calculations of Gliklich and Metson result in $1220 per patient [17].

In the United States the medical spending for AR almost doubled from $6.1 billion in 2000 to $11.2 billion in 2005 [18]. Nasal saline irrigation in AR patients helps to reduce medicine consumption by an average of 2.99% [19]. Thermal water applications present an inexpensive nonpharmacological adjunction as well and could therefore further reduce the medicine consumption and costs.

Thermal water treatment belongs to Balneology. Balneology (lat.* balneum*: bath) is the science of natural curative waters, curative gases, and peloides and their use in the treatment of diseases not only as baths, inhalations, or irrigations but also as drinking cures or mud packs [20]. Thermal inhalations and irrigations are century-old practices and already the Romans appreciated the health-promoting effects of different thermal sources.

According to German regulations, natural curative waters are characterised by a minimum content of 1 g dissolved minerals per liter. The designation “thermal water” requires a temperature of a minimum of 20°C when emerging from the spring [21]. Among the many different types of thermal waters our focus is on sulphurous and radon waters. Sulphurous water and its therapeutical use belong to the oldest forms of balneology. It is said to break disulphide bonds of the mucin and activate breathing and blood circulation and helps to reduce inflammation [20, 22, 23]. Radon is a radioactive gas which emits alpha rays. Its very low content in thermal sources has biopositive effects which stimulate cellular activity [24].

Several studies show significant results in thermal water treatment with inhalation or irrigation treatment [25–29]. Up to now no systematic review or meta-analysis on thermal water application in upper airway diseases exists.

## 2. Materials and Methods

A comprehensive search in the databases of MEDLINE (Medical Literature Analysis and Retrieval System Online), CENTRAL (Cochrane Central Register of Controlled Trials), EMBASE (Excerpta Medica Database), Web of Science, and MedPilot was conducted. In the systematic search the terms “thermal water,” “Spa therapy,” “thermal water inhalations,” and “Spa treatment” were combined with the terms “rhinitis,” “rhinosinusitis,” “allergic rhinitis,” “chronic rhinitis,” and “nasal irrigation” with the Boolean operator “and” in all fields. Furthermore, the terms “Radon Spa therapy,” “Balneotherapy,” “Sulphurous Water,” “Bromide Water,” “Iodic Water,” “Salty Water,” and “Radon Water” were linked through “and” with “rhinitis” or “rhinosinusitis.” No limitation was made in language, publication date, and duration of the study or the demographic data of patients. Literature published up to and including 27 February 2014 was included.

The inclusion criteria for this meta-analysis were as follows: clinical studies conducted with thermal water for the upper airway diseases, allergic, chronic, or acute rhinosinusitis, at least three or more points on the modified JADAD scale [30], and the presence of the complete statistical data sets consisting of mean deviation, standard deviation, and sample size (if appropriate, calculation using standard error or the upper and lower quartile) at defined follow-up time points.

The following outcome parameters were examined in this study: the mucociliary clearance time (MCT), nasal respiratory flow (Flow), nasal resistance (R), immunoglobulin values A, E, G, and M, and adverse events (AE).

## 3. Data Collection and Analysis

The search described above initially resulted in 2113 matches. Duplicates and studies that were either nonclinical or disease-specific not relevant for this analysis were excluded so that the abstracts of 50 remaining studies were examined. Another 15 could be excluded after the perusal of the abstract. 35 studies were investigated in full-text which led to the exclusion of another seven studies due to divergent treatment modalities [22, 31, 32], mismatching disease patterns [33, 34], unavailability [35], or the conduction of the study with animals [36].

The remaining 28 articles were evaluated by two independent reviewers with the modified Jadad Scale by Oremus et al. [30]. It amplifies the original 3-item Jadad Scale [37] consisting of randomization, blinding, and study dropouts by adding inclusion/exclusion criteria, side effects, and statistical methods. Additionally, it features two bonus points for appropriate randomisation method and double-blinding. If this does not apply, these points are deducted. The minimum score is 0 points; the maximum score is 8 points.

We set a minimal score of at least three points to establish a qualitative homogeneity essential for our meta-analysis. Thus another 15 studies were excluded [38–52] and 13 studies remained for our analysis. Four of those are in Italian and nine in English.

**Figure 1:**
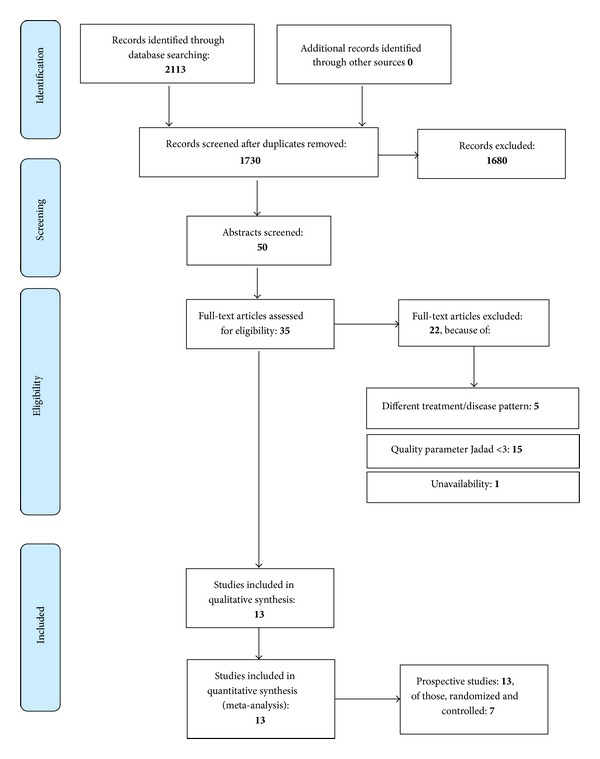
Flow chart. Source: PRISMA 2009 Flow chart [61], augmented with exclusions and types of included studies.

The different thermal waters used in the studies included were pooled concerning their different substances. This resulted in two main groups: sulphurous water with nine studies [25–28, 53–57] and radon water with two studies [29, 58]. Salt-bromine-iodine water [59] and hypermineral chloride sodium water [60] were used once. We also pooled a common thermal water group to compare it to isotonic sodium chloride solution (ISCS) and placebo.  Figure 1 shows a flowchart of the literature identification process. Tables 1 and 2 show the included studies in a systemic overview.

### 3.1. Statistical Methods

**Figure 2:**
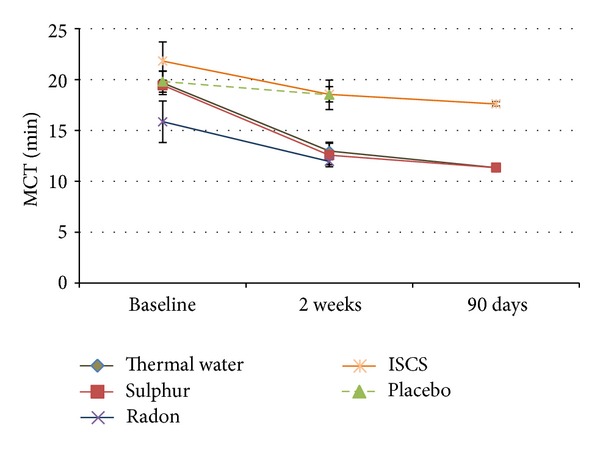
Mucociliary clearance time.

**Figure 3:**
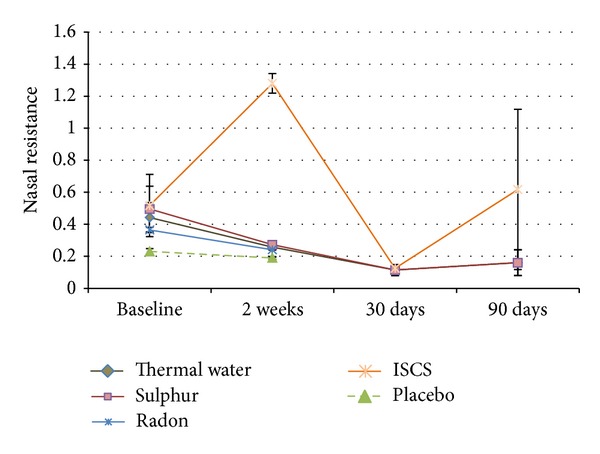
Nasal resistance.

**Figure 4:**
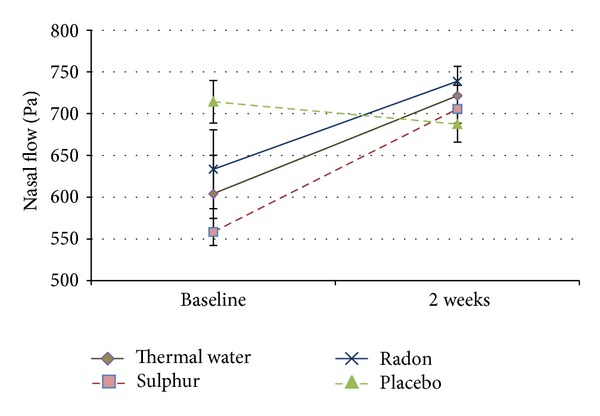
Nasal flow. Dotted lines present single studies.

**Figure 5:**
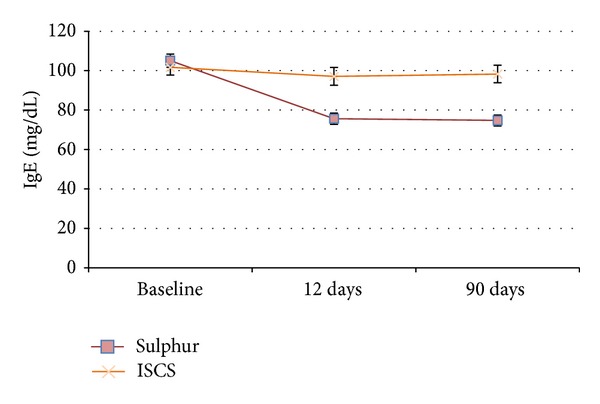
IgE in serum.

The statistical calculations were performed using the statistic software Comprehensive Meta-Analysis Version 2.2.064 (Biostat, Inc.). The study values for the identified outcome parameters were sorted according to the time of measurement (baseline, 12 days, 2 weeks, 30 days, and 90 days). Studies with the same parameter and different (follow-up) times of measurement, of 12 and 14 days, were combined to one-time measurement (2 weeks). All points of measurements of the different studies were summarised and analysed using the random-effect model. The mean and, respectively, the standard error of the mean are depicted in the figures (Figures 2–5). In the following analysis we assumed a significance if the *P* value was less than or equal to 5% (*P* ≤ 0.05). For clarification, significant improvements/changes are marked with  * in Tables 4–7. For some identified parameters data were available from one study only and therefore had to be excluded from the meta-analysis but are, for the sake of completeness, included in the figures with an interrupted line.

## 4. Results

In total, 13 studies published between 1998 and 2013 have been included in this analysis.

All forms of applications were pooled. The specific form of application and the number of patients is depicted in Table 3. An aerosol therapy is categorised under inhalations. Altogether, 430 patients received irrigations and 557 patients inhalations.

### 4.1. Study Design

All studies feature a prospective study design. Five studies are randomised, controlled, and double-blind [25–28, 54]; other three studies are randomised and controlled [56, 59, 60]; one study is controlled and double-blind [29]; one is only double-blind [55]. The remaining studies are not randomised, blinded, or controlled [53, 57, 58]. ISCS was used for the control groups, drinking water or distilled water for placebo groups. The duration of the studies varies between 12 days and 6 months.

### 4.2. Selection of Patients

A total number of 840 patients aged between 2 and 100 years took part in these studies, 510 of them received an application with thermal water, 285 were treated with ISCS [25–28, 56, 59], 20 inhaled drinking water [29], and 25 inhaled distilled water [54].

### 4.3. Mucociliary Clearance Time

MCT was examined in seven studies with 422 patients in total (Table 4). Thermal water (radon, sulphur, and salt-bromine-iodine) applications showed a significant improvement of MCT compared to baseline at both points of measurement (Figure 2). The measurement for ISCS compared to baseline showed no significance after two weeks but after 90 days in the followup. Only one study was conducted with placebo which did not show any significance neither did radon water applications (*P* = 0.059). Sulphurous water applications showed significant lower values after two weeks compared to the baseline value (*P* < 0.01) and are also significant lower after 90 days compared to the baseline values (*P* < 0.01).

In an internal comparison between the ISCS group and the sulphurous water group we had nonsignificant initial situations (*P* = 0.211), but after 2 weeks and 90 days the outcome differed significantly (*P* < 0.01). The ISCS and the radon group already differed significantly in baseline values (*P* < 0.05) and had almost parallel curves.

Figure 2 illustrates all values calculated in the meta-analysis and noted in Table 4.

### 4.4. Nasal Resistance

Nasal resistance was measured in six of the included trials with a total number of 347 patients (Table 5). Thermal water treatment was not significant after two weeks but after 30 and 90 days compared to baseline. ISCS treatment showed significance after two weeks and 30 days compared to baseline, but after 90 days there was no significance. This graph (Figure 3, ISCS graph) showed a very erratic curve due to the very heterogeneous study design and the different points of measurement of the three studies included. Both the treatment with placebo and the treatment with radon water showed significance after two weeks. The treatment with sulphurous water showed significance after 30 (*P* < 0.01) and 90 days (*P* < 0.05) but not after two weeks (*P* = 0.118).

### 4.5. Nasal Flow

The nasal flow was specified in three of the included trials with 117 patients (Table 6). All of these patients received inhalation and aerosol therapy.  Figure 4 shows all included studies, using this outcome parameter, the dotted lines for placebo and sulphur indicate single studies. Compared to baseline, the combined thermal water group (*P* < 0.05), as well as the radon (*P* < 0.05) and the sulphurous water group (*P* < 0.01), showed significant improvement after two weeks, whereas drinking water application (*P* = 0.425) showed no improvement or significance.

### 4.6. Immunoglobulins E, A, G, and M

Immunoglobulin concentrations in the blood were examined in two of the included trials [25, 26] with a total number of 180 patients. Both studies compared sulphurous water treatment to ISCS treatment. Distribution of the number of patients (90 patients per group) was equal in both studies.

### 4.7. IgE

Figure 5 illustrates the meta-analysis outcome for IgE. Both groups started from a comparable initial position. Sulphurous water treatment rose significantly (*P* < 0.01) at both measurement points 12 and 90 days compared to baseline in contrast to ISCS treatment which was neither significant after 12 days (*P* = 0.442) nor after 90 days (*P* = 0.567).

### 4.8. IgA, G, and M

No significant differences could be revealed in the analysis of the immunoglobulins A, G, and M, neither beyond the subgroups between ISCS and sulphur nor in the individual groups between the baseline and the maximal treatment duration of 90 days.

### 4.9. Adverse Events

All adverse events that occurred during the studies in the entire patient population were extracted and illustrated in forest plots. In total, 19 patients out of 840 treated patients suffered from study related adverse events. All adverse events occurred under the treatment with sulphurous water: 13 patients experienced mild nasal irritation and a sensation of burning after application and five suffered from very limited epistaxis, one from an aggravation of the symptoms, and one from dermatological hypersensitivity. No adverse events are reported for the treatment with another thermal water, ISCS, or placebo.

**Figure 6:**
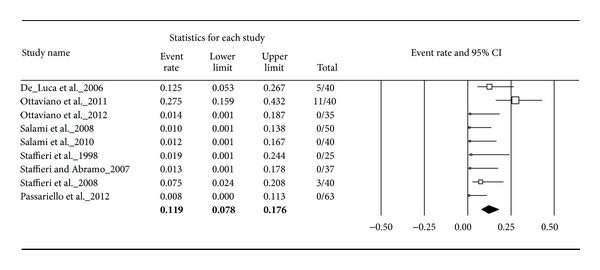
Adverse events for sulphurous water.

For sulphurous water, 19 adverse events occurred in a total group of 370 patients. This led to an adverse event rate of 11.9%. The assumed rate of adverse events ranged from 7.8 to 17.6% (Figure 6).

**Figure 7:**
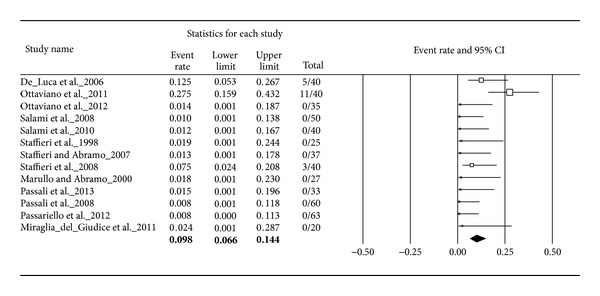
Adverse events for thermal water.

By pooling all thermal water subgroups we received a total number of 510 treated patients with 19 adverse events. This led to an adverse event rate of 9.8% with an assumed range from 6.6 to 14.4% (Figure 7).

## 5. Discussion

This review and its appertaining meta-analysis is the first systematic approach to thermal water treatment in upper respiratory tract diseases. For the identified outcome parameters some significant improvements could be found in the treatment with thermal water irrigation and inhalation.

In order to ensure methodological quality of the included trials, two independent reviewers applied the modified Jadad Scale to every study with a minimal score of 3.

Further this meta-analysis is calculated by the “random effects” model, which takes possible heterogeneity more into consideration than the “fixed effect” model. The confidence intervals are broader and thus capture the true value of the meta-analysis. Where relative overestimation of smaller studies can result in greater inaccuracy, this is a more conservative and cautious estimation. It constitutes a higher risk for bias of the results [63].

In addition, we pooled the clinical pictures of allergic, acute, and chronic rhinosinusitis as well as studies with children, adults, and elderly people. In two studies patients with minimally invasive functional endoscopic sinus surgery (FESS) before treatment were included. This can be assumed as selection bias for this meta-analysis. Furthermore, only published studies were included in this meta-analysis. Thus, publication bias may occur.

Besides irrigation, also inhalation therapy was used in the different studies. Both treatments reduced inflammatory mediators in nasal secretions [64]. Nasal irrigation had a direct physical cleansing effect by flushing out thick mucus, crusts, debris, allergens, and air pollutants [65]. In a review by Hermelingmeier et al. the conclusion drawn is that there is no clear data available naming the most advantageous form of application in nasal saline irrigation [19].

The present meta-analysis shows a significant advantage of mucociliary clearance changes with thermal water in comparison to isotonic saline solution. This leads to a more detailed view of the results of thermal water applications found in this meta-analysis.

The MCT parameter comprises the best set of data in our meta-analysis with seven included studies and 422 patients. Mucociliary clearance is an important defence mechanism for both upper and lower airways and “its impairment[…] predisposes to chronic infection of the nose, paranasal sinuses and the respiratory tree” [66]. The average MCT values range below 15 minutes with a test duration of less than 1 hour [67]. In this meta-analysis a significant improvement of the mucociliary clearance could be determined in thermal water applications. Hereby, the transport time could be reduced from 19.67 minutes initially to 12.98 minutes after two weeks and to 11.34 minutes after 90 days. Especially the application of sulphurous water showed a high significance (*P* < 0.001) after the two-week treatment period. At the same time the treatment with radon thermal water (*P* = 0.059) only just lacked a significant improvement (*P* = 0.059).

The literature available for these two subgroups was 5 : 2 with only 60 patients in the radon group which might have had an influence on the significance. In contrast to the thermal water group, the ISCS group only showed significance after 90 days of treatment. In turn the literature available was quite strong with 4 studies and a total of 205 patients.

Based on Marullo's study only it was not possible to conduct a meta-analysis and draw a valid conclusion on the use of placebo [29]. The meta-analysis of the mucociliary clearance time showed a significant benefit especially of the pooled thermal water treatment and sulphurous water over ISCS.

The “nasal resistance is the resistance offered by the nasal cavity to inspired air” [68] and it is measured in Pascal (Pa). All studies used for this analysis already resulted in significant changes after two weeks of treatment with radon thermal water. ISCS differed significantly after two weeks and after 30 days of treatment. The meta-analysis revealed significant variations in the three pooled ISCS studies. Especially the study of Salami et al. displayed a high deviation from the baseline of 13.1 Pa, which was reduced to 1.28 Pa after two weeks and remained rather high after 90 days of treatment with 1.12 Pa.

Opposed to these findings were those of the two other studies of this pool [27, 28], which began with much lower baseline values of 0.14 and 0.17 Pa and had different followups. Therefore these led to an unsteady curve and limited the possibility of a serious interpretation of the healing process. Nevertheless, each of these three studies showed a reduction of the nasal resistance.

The treatment with sulphurous water showed good results throughout the whole treatment period of 90 days. The results were even better after 30 and 90 days than at the beginning, which allows for the assumption that a more permanent improvement is gained here.

Based on this meta-analysis we can assume that radon water application shows significant improvement in nasal flow. The data is quite limited with the results of only one study for placebo and one for sulphurous treatment, so that we cannot compare it to the pooled results.

The use of radon thermal water as well as the entire thermal water subgroup showed a significant improvement in the nasal flow after two weeks of treatment.

In our meta-analysis of IgE, sulphurous water treatment was highly significant after 12 and 90 days. ISCS treatment showed no significance. The present IgE results were measured in patients with chronic inflammatory conditions, where eosinophil cells in the mucus are increased [69]. Reduction of eosinophil cells after thermal water treatment was also reported in Passali et al. [58] and significantly decreased in Staffieri and Abramo [55]. Hypereosinophilia is related to high levels of serum concentrations of IgE [26, 70]. The IgE concentration which decreased significantly after the application with sulphurous water confirmed beneficial effects on chronic inflammatory disorders. Sulphurous water helps to clean the nasal mucosa from irritations and reduces immune responses at a local level [25, 71]. These results support the assumption that sulphurous water has an anti-inflammatory effect.

IgA, IgG, and IgM values in the blood did not increase significantly neither with sulphurous water nor with ISCS. Unfortunately the literature available on the secretory IgA, which is secreted across the mucosa and plays a significant role in specific immune defence by preventing or blocking the adhesion of bacteria and defending the mucous membranes from common infection [72, 73], was not sufficient for a statistical analysis. Similarly, comparable studies investigating the IgM and IgG in the mucosa were missing.

Generally speaking, thermal water application is a safe treatment. Adverse events occurred in 19 out of 510 thermal water treatments and mainly consisted of mild nasal irritation, a sensation of local burning after application, and very limited epistaxis. All of these adverse events occurred under the treatment with sulphurous water. Neither for radon water, ISCS, nor placebo treatment adverse events were reported. It should be noted that both the studies by Staffieri et al. [56] and Ottaviano et al. [28] were conducted in a postoperative setting, which makes the occurrence of such adverse events more likely. Further, the study by De Luca et al. [53] was conducted with elderly people between 72 and 100 years.

## 6. Conclusion

Nasal application of thermal water results in a significant improvement of MCT, nasal flow, nasal resistance, and IgE concentration. The systematic review and the meta-analysis demonstrate an advantage of thermal water treatment over isotonic saline solution and placebo. Even though this aspect needs to be investigated further with randomised controlled trials in bigger cohorts and longer follow-up periods, it was shown that the application with thermal water can serve as additional nonpharmacological alternative.

## Figures and Tables

**Table 1:** Included studies.

Author	Year	Study design	Thermal water/control	Application	Adverse events	MCT	Flow	Resistance	IgA	IgE	IgG	IgM
De Luca et al. [53]	2006	Prospective, nonrandomized, noncontrolled	Sulphur/—	Inhalation 1x daily 10 min for 12 days	X	X						

Marullo and Abramo [29]	2000	Prospective, nonrandomized, controlled, double-blind	Radon/Tap water	Inhalation 10 min, Aerosol 10 min, 1x daily for 12 days	X	X	X	X				

Miraglia del Giudice et al. [60]	2011	Prospective, randomized, controlled	Hypermineral chloride sodium water/ISCS	Aerosol 15 days a month for 3 months/ ISCS daily for 3 months	X							

Ottaviano et al. [27]	2012	Prospective, randomized, controlled, double-blind	Sulphur-Arsenic-Ferrous water diluted to 10% solution with distilled water/ISCS	Irrigation 1x daily 20 mL for 1 month	X			X				

Ottaviano et al. [28]	2011	Prospective, randomized, controlled, double-blind	Sulphur-Salt-Bromine-Iodine/ISCS	Irrigation 4x daily 5 mL for 1 month	X			X				

Passali et al. [58]	2013	Prospective, nonrandomized, noncontrolled	Radon/—	Inhalation, Aerosol, each 10 min 1x daily for 14 days	X	X	X	X				

Passali et al. [59]	2008	Prospective, randomized, controlled	Salt-Bromine-Iodine/ISCS	Inhalation 10 min, Aerosol 10 min, Irrigation 5 min 1x daily for 14 days	X	X						

Passariello et al. [57]	2012	Prospective, nonrandomized, noncontrolled	Sulphate-Sodium-Chloride/—	Aerosol 15 min 1x daily for 15 days	X							

Salami et al. [26]	2010	Prospective, randomized, controlled, double-blind	Sulphur/ISCS	Inhalation, 10 min Irrigation 6 min 1x daily for 12 days	X	X		X	X	X	X	X

Salami et al. [25]	2008	Prospective, randomized, controlled, double-blind	Sulphur/ISCS	Inhalation 10 min 1x daily for 12 days	X	X			X	X	X	X

Staffieri et al. [56]	2008	Prospective, randomized, controlled	Sulphur-Arsenic-Ferrous water diluted to 10% solution with distilled water/ISCS	Irrigation 4x daily 20 mL for 6 months	X							

Staffieri and Abramo [55]	2007	Prospective, nonrandomized, noncontrolled, double-blind	Sulphur-Arsenic-Ferrous water/—	Inhalation, Aerosol each 10 min, 1x daily for 12 days	X	X	X	X				

Staffieri et al. [54]	1998	Prospective, randomized, controlled, double-blind	Sulphur-Salt-Bromine-Iodine/distilled water	Inhalation, 10 min, Aerosol 10 min 1x daily for 12 days	X							

**Table 2:** Systematic presentation of the studies included.

Author	Year	Jadad score	Number of patients thermal water/control	Age span/average age	Duration of treatment	Measurement time points	Inclusion criteria	Exclusion criteria
De Luca et al. [53]	2006	3	40/—	74–100/86,5	12 days	Baseline Follow-Up: Day 12	(i) Presence of chronic rhinitis or chronic rhinosinusitis (ii) Allergic or vasomotor nasal hyperactivity (iii) Chronic laryngitis (iv) Chronic pharyngitis	(i) Acute pathologies in ENT region (ii) Steroids, mucolytics, antihistamine, NSAIDs, vasoconstrictive drugs or antibiotics in the last 30 days (iii) Autoimmune disease (iv) Patients with malignant neoplasms, surgical intervention, and/or radio chemotherapy

Marullo and Abramo [29]	2000	3	27/20	15–81/52.4	12 days	Baseline Follow-Up: Day 12	(i) At least 3 confirmed episodes of sinonasal infection in the previous 12 months (ii) Evidence of chronic sinonasal inflammation at objective otorhinolaryngologic evaluation	(i) Vasoconstrictive drugs, local and systemic steroids, NSAIDs, antihistamine, and mucolytics in the last 2 months (ii) Patients who for various reasons were not able to ensure the conclusion of the study (iii) Patients who present anterior rhinoscopy

Miraglia del Giudice et al. [60]	2011	5	20/20	6–14/	15 days a month for 3 months	Baseline Follow-Up: Week 12, 14	(i) Age from 6 to 14 (ii) History of seasonal moderate to severe allergic rhinitis for at least 2 years (iii) Positive skin prick test to *Parietaria* pollen (iv) History of mild intermittent asthma	(i) Antihistamines, intranasal, bronchial, or systemic corticosteroids, cromolyn sodium, and leukotriene modifiers in the previous 6 weeks (ii) sinus and/or upper or lower respiratory tract infection, persistent asthma, nasal surgery within the last year, respiratory tract abnormalities, and systemic diseases

Ottaviano et al. [27]	2012	5	35/35	18–65/ not specified	12 weeks	Baseline Follow-Up: Day 30, 90	(i) Age from 18 to 65 (ii) Nonallergic chronic rhinitis (iii) Cigarette smoking habit for at least 5 years	Autoimmune diseases, cystic fibrosis, and diabetes

Ottaviano et al. [28]	2011	8	40/40	18–65/ not specified	1 month	Baseline Follow-Up: Day 30	(i) Age from 18 to 65 (ii) Nonallergic chronic rhinosinusitis	Autoimmune diseases, cystic fibrosis, and diabetes

Passali et al. [58]	2013	3	33/—	12 years and older/ not specified	14 days	Baseline Follow-Up: Day 14	(i) Nasal obstruction evaluated by a 10-point Visual Analog Scale (1: nasal airways completely free; 10: nasal airway completely blocked) higher than 7 in the previous 2 months (ii) Chronic rhinosinusitis, persistent allergic rhinitis, vasomotor rhinitis with inferior turbinate hypertrophy	(i) Acute viral rhinitis (ii) Obstructive polyposis (iii) Nasal steroid, vasoconstrictive drug therapies or systemic NSAIDs, oral steroids and mucolytic treatment in the previous 2 months

Passali et al. [59]	2008	3	60/60	15–65/not specified	14 days	Baseline Follow-Up: Day 14	Chronic rhinosinusitis with/or nasal polyposis of I second degree of the Lund-Mackay-Classification [62]	Not specified

Passariello et al. [57]	2012	3	60/—	2–12/3.4 ± 1	15 days	Baseline Follow-Up: Day 15	(i) Age from 2 to12 (ii) CRS (iii) One or more of the following sinonasal symptoms: nasal discharge, congestion, obstruction, postnasal drip, daytime cough, and foul breath (iv) Failed courses of antibiotics, saline irrigation, nasal steroids, or antihistamine (v) Persistent symptoms for ≥1 month	(i) Steroids, nonsteroidal anti-inflammatory drugs, antihistamines, and vasoconstrictors in the previous 4 weeks (ii) Primary diagnosis of obstructive sleep apnea syndrome caused by tonsillar hyperplasia (iii) Chronic diseases, immunodeficiency, and neurological impairment (iv) Varicose veins of the nasal septum, and suspect ciliary abnormalities (v) Previous sinonasal surgery (vi) Malformation of the upper airway sinonasal osteogenesis, tumors, and obstructive lesions (vii) History of facial trauma that distorted the sinus anatomy

Salami et al. [26]	2010	5	40/40	26–58/46.4	12 days	Baseline Follow-Up: Day 12, 90	CRS without polyps	(i) Immunostimulant or immunosuppressive agents in the previous 6 months (ii) Genetic and congenital condition: cystic fibrosis and primary ciliary dyskinesia (iii) Nasal polys (iv) Positive allergy testing (v) Anatomic abnormalities (severe septal deviation among others) (vi) Acquired mucociliary dysfunction (vii) Neoplasms (viii) Acute contemporary bacterial and/or viral rhinosinusitis, middle ear, and respiratory tract (ix) Bronchopulmonary disease (x) Nasal trauma (xi) Smoker (xii) Previous nasal and sinus surgery

Salami et al. [25]	2008	7	50/50	6–14/9	12 days	Baseline Follow-Up: Day 12, 90	At least 3 acute episodes of upper respiratory tract infections in the last year	(i) Immunostimulant or immunosuppressive agents in the previous 6 months (ii) Previous adenoidectomy and/or tonsillectomy (iii) Anatomic anomalies (iv) Other acute infections (v) Allergic rhinitis (vi) Congenital immunodeficiency's (vii) Pulmonary disease

Staffieri et al. [56]	2008	5	40/40	18–65/not specified	6 months	Baseline Follow-Up: Day 30, 90, 180	(i) Age from 18 to 65 (ii) CRS not responding to medical treatment (iii) No contraindications to general anaesthesia and FESS	(i) Autoimmune disease (ii) Cystic fibrosis (iii) Diabetes (iv) Sinonasal inverted papilloma or sinonasal malignancy

Staffieri and Abramo [55]	2007	4	37/—	40/not specified	12 days	Baseline Follow-Up: Day 12	(i) At least 3 confirmed episodes of sinonasal infection in the previous 12 months (ii) Evidence of chronic sinonasal inflammation at otorhinolaryngologic endoscopic evaluation	Nasal steroid, vasoconstrictive drug therapies or systemic NSAIDs, and steroid or mucolytic treatments in the previous 2 months

Staffieri et al. [54]	1998	3	25/25	18–83/50.5	12 days	Baseline Follow-Up: Day 12	Chronic rhinopharyngotubaric inflammation	Not specified

**Table 3:** Type of application.

Application	Inhalation	Inhalation + aerosol	Irrigation	Inhalation + irrigation	Inhalation + aerosol + irrigation	Aerosol
Number of patients	140	117	230	80	120	100
Study	De Luca et al. [53] Salami et al. [25]	Marullo and Abramo [29] Passali et al. [58] Staffieri and Abramo [55] Staffieri et al. [54]	Ottaviano et al. [27] Ottaviano et al. [28] Staffieri et al. [56]	Salami et al. [26]	Passali et al. [59]	Miraglia del Giudice et al. [60] Passariello et al. [57]

**Table 4:** Results of MCT.

	Patients	MCTt	CI 95%	*P* value
Thermal water (sulphur + salt-bromine-iodine + radon)				
Baseline	265	19.67020883	[17.40; 21.94]	
2 weeks	265	12.98258754	[11.34; 14.63]	<0.01*
90 days	90	11.34482778	[10.91; 11.78]	<0.01*
ISCS				
Baseline	137	21.81138859	[18.19; 25.05]	
2 weeks	137	18.54655442	[17.09; 20.01]	0.107
90 days	90	17.60438587	[17.17; 18.04]	<0.05*
Placebo				
Baseline	20	19.8	[17.78; 21.82]	
2 weeks	20	18.5	[15.65; 21.35]	0.465
Radon				
Baseline	60	15.84974132	[11.85; 19.85]	
2 weeks	60	11.95305164	[11.33; 12.58]	0.059
Sulphur				
Baseline	205	19.43087224	[18.89; 19.97]	
2 weeks	205	12.57267757	[10.30; 14.85]	<0.01*
90 days	90	11.34482778	[10.91; 11.78]	<0.01*

*Significant in comparison to baseline (*P* < 0.05).

**Table 5:** Results of nasal resistance.

	Patients	NasRes	CI 95%	*P* value
Thermal (sulphur +radon)				
Baseline	212	0,442260114	[0.26; 0.62]	
2 weeks	137	0.257467503	[0.22; 0.30]	0.051
30 days	66	0.113901251	[0.05; 0.18]	<0.01*
90 days	64	0.159927083	[0.003; 0.32]	<0.05*
ISCS				
Baseline	115	0.516633	[0.13; 0.90]	
2 weeks	40	1.28	[1.16; 1.40]	<0.01
30 days	62	0.124408602	[0.10; 0.15]	<0.05
90 days	68	0.617283393	[−0.36; 1.60]	0.851
Placebo				
Baseline	20	0.23	[0.19; 0.27]	
2 weeks	20	0.19	[0.17; 0.21]	<0.05*
Radon				
Baseline	60	0.364606147	[0.33; 0.40]	
2 weeks	60	0.240700629	[0.15; 0.33]	<0.05*
Sulphur				
Baseline	152	0.495021367	[0.22; 0.77]	
2 weeks	77	0.272182942	[0.26; 0.29]	0.118
30 days	66	0.113901251	[0.05; 0.18]	<0.01*
90 days	64	0.159927083	[0.003; 0.32]	<0.05*

*Significant in comparison to baseline (*P* < 0.05).

**Table 6:** Results of nasal flow.

	Patients	NasFlow	CI 95%	*P* value
Thermal (sulphur + radon)				
Baseline	97	604.1	[513.68; 694.45]	
12–14 days	97	721.5	[697.18; 745.84)	<0.05*
Placebo				
Baseline	20	714.3	[664.13; 764.47]	
12–14 days	20	687.5	[644.79; 730.21]	0.425
Radon				
Baseline	60	633.4	[540.95; 725.90]	
12–14 days	60	738.8	[703.64; 773.89]	<0.05*
Sulphur				
Baseline	37	558.4	[526.53; 590.27]	
12–14 days	37	705.6	[671.86; 739.34]	<0.01*

*Significant in comparison to baseline (*P* < 0.05).

**Table 7:** Results of IgE.

	Patients	IgE	CI 95%	*P* value
Sulphur				
Baseline	90	105.11	[98.53; 111.69]	
12 days	90	75.65	[70.13; 81.18]	<0.01*
90 days	90	74.79	[69.38; 80.19]	<0.01*
ISCS				
Baseline	90	101.69	[94.03; 109.35]	
12 days	90	97.10	[88.22; 105.98]	0.442
90 days	90	98.30	[89.60; 107.00]	0.567

*Significant in comparison to baseline (*P* < 0.05).

## Abbreviations

AE: Adverse events
AR: Allergic rhinitis
ARIA: Allergic rhinitis and its impact on asthma
ARS: Acute rhinosinusitis
CRS: Chronic rhinosinusitis
EPOS: European Position Paper on Rhinosinusitis and Polyps
FESS: Functional endoscopic sinus surgery
Flow: Nasal respiratory flow
Ig: Immunoglobulin
ISCS: Isotonic sodium chloride solution
MCT: Mucociliary clearing time
NAR: Nonallergic rhinitis
Pa: Pascal
R: Resistance
RURT: Recurrent upper airway infections.
